# Rosai-Dorfman: Rare Manifestations of a Rare Disease

**DOI:** 10.7759/cureus.36673

**Published:** 2023-03-25

**Authors:** Caroline E Moore, James H Flint, Kevin M Taniguchi, Preston S Gable

**Affiliations:** 1 Internal Medicine, Navy Medicine Readiness and Training Command San Diego, San Diego, USA; 2 Orthopedic Surgery/Orthopedic Oncology, Navy Medicine Readiness and Training Command San Diego, San Diego, USA; 3 General Surgery, Navy Medicine Readiness and Training Command San Diego, San Diego, USA; 4 Hematology and Medical Oncology, Navy Medicine Readiness and Training Command San Diego, San Diego, USA

**Keywords:** environmental exposures, extranodal involvement, mapk1, histiocytosis, rosai-dorfman disease

## Abstract

Rosai-Dorfman disease (RDD) is an exceedingly rare non-Langerhans cell histiocytosis of uncertain etiology that most commonly presents in children as self-limited, painless, massive cervical lymphadenopathy. However, extranodal disease occurs in 43% of cases and has a wide range of phenotypic presentations. The pathogenesis has not been clearly understood in the literature and coupled with a wide range of clinical manifestations, early diagnosis and initiation of an appropriate treatment modality have been challenging.

Herein, we describe a cohort of five cases that occurred at the same institution within a 12-month period. These cases highlight unique and atypical presentations of an already rare disease, outline the varying and tailored diagnostic and therapeutic approaches, and propose a novel environmental predisposing factor given the exceptionally high incidence at our institution over a short period of time. We emphasize the need for further investigation of predisposing factors and to discern targeted therapies that may offer benefits*.*

## Introduction

Rosai-Dorfman disease (RDD) is a rare non-Langerhans cell histiocytosis with heterogeneous phenotypic presentations. It is characterized by the infiltration of activated histiocytes within nodal and extranodal tissues as well as a variable occurrence of emperipolesis [1]. The pathophysiology of RDD has not been well-defined, but it may occur in association with neoplastic, infectious, or autoimmune diseases [2]. It is estimated that approximately 100 new cases occur in the United States each year, most commonly presenting as bulky, bilateral cervical adenopathy in children and young adults [1,2]. Extranodal disease occurs in approximately 43% of cases, including the skin, nasal cavity, and gastrointestinal system while central nervous involvement occurs in <5% and bone involvement occurs in <10% of cases [2-4]. It is even rarer for bony manifestations to occur in isolation without associated skin or lymph node involvement [3,4]. RDD is a histopathologic diagnosis, and therapeutic modalities vary according to patient-specific factors and affected organs. Many cases are self-limited, but surgery, immunosuppressants, corticosteroids, and chemotherapy are described as treatment modalities [5].

The cases presented herein highlight the heterogeneity of this disease, as each of these presentations is extranodal and atypical. These cases also raise the question of an environmental link to the pathogenesis of RDD, given the uncharacteristically high incidence of RDD in our locoregional population in such a short period of time. Each case illustrates unique obstacles to diagnosis and the varying approaches to treatment. In doing so, this case series provides valuable insight and adds to the knowledge base of this rare disease.

## Case presentation

Case 1

**Figure 1:**
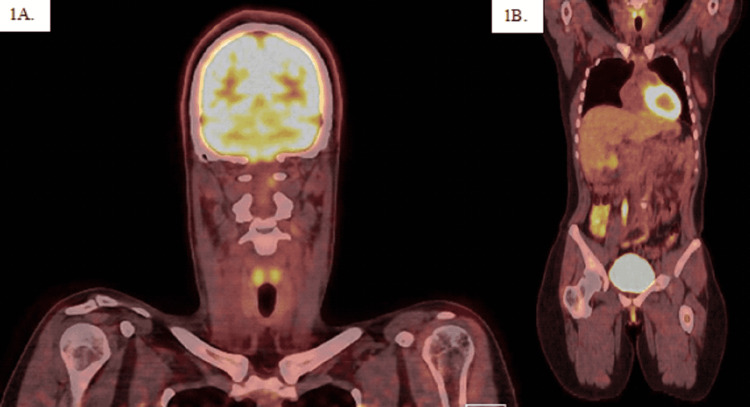
Rosai-Dorfman disease involving the left C1-C2 nerve root Positron emission tomography-computed tomography showed mild hypermetabolic activity within a nodular soft tissue density at the left C1-C2 level (A.) without systemic involvement (B.)

A previously healthy, 22-year-old female on active duty in the Navy presented with the chief complaint of painless progressive decrease in visual acuity of the right eye (OD). The patient was recently married and desired children. The remainder of the history was unremarkable. Due to her decreased visual acuity, the patient was evaluated by an optometrist and found to have a visual acuity of 20/20 in her left eye and 20/60+ in her right eye and was subsequently prescribed corrective lenses, which achieved best-corrected visual acuity (BCVA) to 20/50 in her right eye. Several months later, however, the patient failed an occupational eye exam while aboard a ship and was referred to ophthalmology. A brain magnetic resonance imaging (MRI) was performed revealing enhancing infiltrative lesions in the optic nerve, brain, and spinal cord. She was admitted for an expedited evaluation, which included a lumbar puncture and a bone marrow biopsy out of concern for a possible demyelinating or lymphoproliferative disease based on the radiographic appearance of the lesions on MRI. Each of these studies was non-diagnostic and other laboratory diagnostics, including anti-neutrophil cytoplasmic antibody (ANCA) levels, were negative. A follow-up MRI of the brain and spine four months later demonstrated an infiltrative, enhancing process involving the optic canals, lesser sphenoid wings, left internal auditory canal, and left C2 nerve root. A nasopharyngeal biopsy was attempted, but the results were non-diagnostic. The patient’s vision continued deteriorating, developing decreased visual acuity in the left eye (20/30 with a color deficit). Positron emission tomography-computed tomography (PET/CT) showed mild hypermetabolic activity within a nodular soft tissue density at the left C1-C2 level without systemic involvement (Figures 1A, 1B). Open excisional biopsy was pursued of this intradural, extramedullary mass with neurosurgery. The histopathology findings were consistent with Rosai-Dorfman disease, demonstrating sheets of large histiocytes with emperipolesis, which were positive for CD68 and S100 and negative for CD1a. Neurology prescribed high-dose corticosteroids for several months, but unfortunately, the patient experienced continued deterioration of her vision and new onset left-sided tinnitus. She was subsequently referred to medical oncology for the initiation of cladribine. She completed six cycles of cladribine without further loss of vision or hearing, and possibly improvement of her visual fields per ophthalmology [2]. Surveillance MRI showed a positive treatment response (but not a complete response), with some continued enhancement along the left optic nerve sheath. Next-generation sequencing of her tumor showed overexpression of MET by mRNA. However, the patient decided to leave the local area and transition her care for increased family support. The patient was ultimately referred to an organization that specializes in intra-arterial chemotherapy for direct administration to the optic nerves.

Case 2

**Figure 2:**
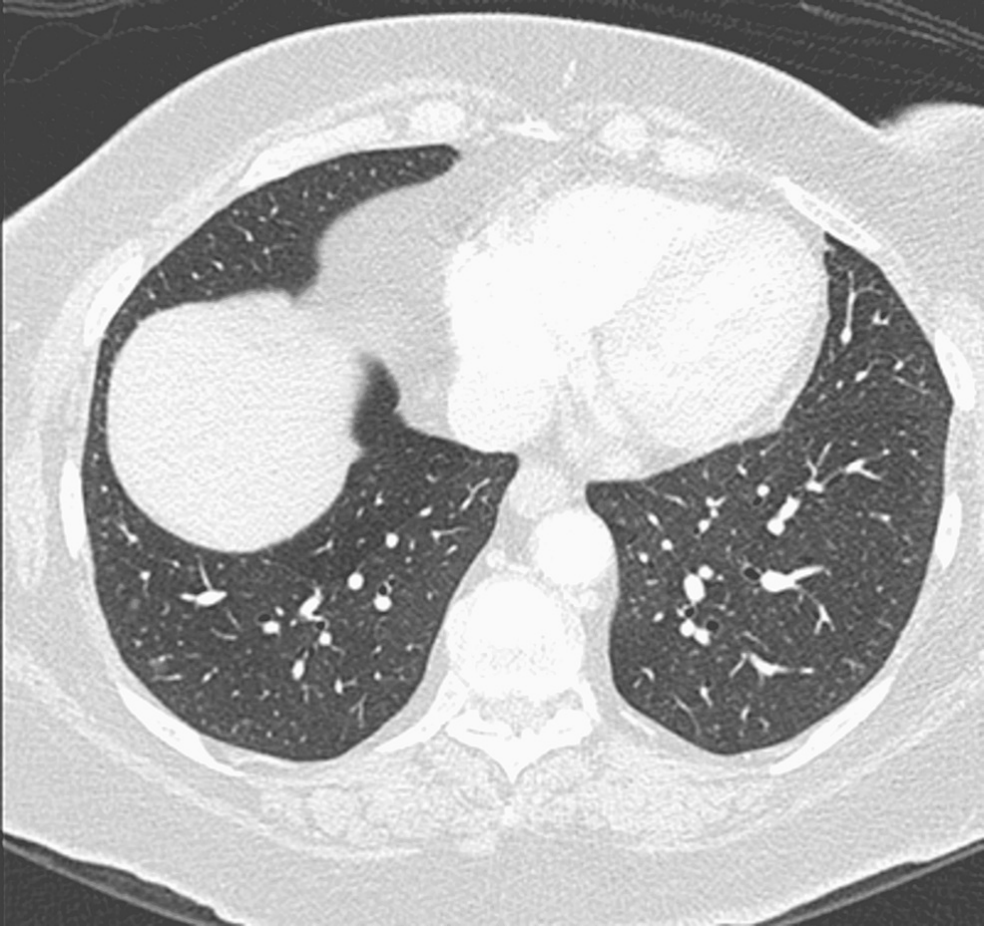
Systemic involvement of Rosai-Dorfman disease of the breast Computed tomography with contrast (a part of positron emission tomography-computed tomography scan (PET-CT)) exhibited small, low-density, nodular, and ground-glass opacities at both lung bases, thought to be representative of Rosai-Dorfman disease.

A 66-year-old female with a history of hypertension and type 2 diabetes mellitus presented to the breast health clinic due to abnormal breast imaging. The patient received a screening mammogram, which identified a 1.9 cm irregular mass in the left upper quadrant of the left breast. She denied feeling a lump in her breast or any other suspicious findings, fevers, weight loss, or night sweats. Physical examination did not reveal any palpable adenopathy. Initial ultrasound-guided core biopsy revealed fibrous and adipose tissue with lymphoplasmacytic infiltration consistent with a reactive or infectious process. A repeat mammogram re-demonstrated the mass, and due to the high suspicion of malignancy, an excisional biopsy was sought. The biopsy revealed effaced histiocytes, plasma cells, emperipolesis, and positive S100 staining, consistent with the diagnosis of Rosai-Dorfman disease. To evaluate for systemic disease, a positron emission tomography-computed tomography scan (PET-CT) was performed and revealed several small, low-density nodular and ground-glass opacities at both lung bases and were thought to be part of the patient’s RDD presentation (Figure 2). Corticosteroid therapy with prednisone 50 milligrams daily for 10 days was prescribed. A repeat non-contrast chest CT two months later revealed persistence of the scattered ground-glass nodules. She was referred to pulmonology but declined this consultation as well as a follow-up chest CT, as she was completely asymptomatic.

Case 3

**Figure 3:**
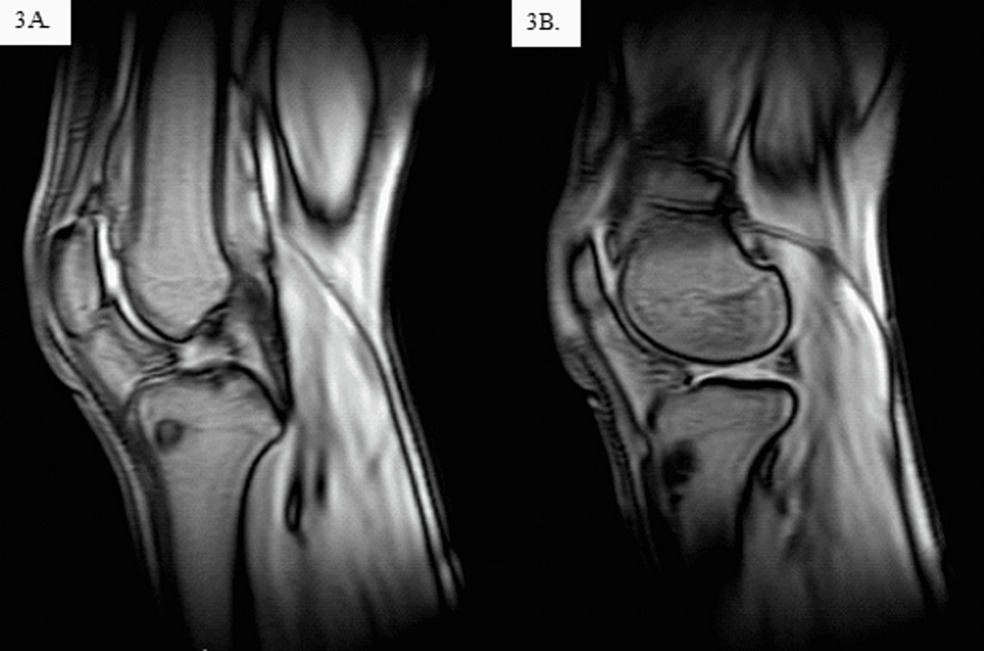
Rosai-Dorfman disease involving the proximal left tibia Magnetic resonance imaging (MRI) revealed two well-circumscribed intramedullary lesions within the left proximal tibia without cortical disruption

 A 22-year-old male on active duty in the Navy presented with a one-year history of left knee pain, first noted after he injured his knee stepping into a hole. Despite physical therapy and a course of anti-inflammatories, his pain persisted, and he was referred to orthopedics. On physical examination, there were no adenopathy or skin lesions and no restriction to motion or strength. Radiographs of his left knee were normal in appearance, with no evidence of fracture. An MRI with and without contrast demonstrated two well-circumscribed, contrast-enhancing, intramedullary lesions within the proximal tibia (Figures 3A, 3B). There were no soft tissue masses, periosteal reaction, cortical destruction, or other aggressive features. Both a bone scan and screening CT chest, abdomen, and pelvis demonstrated no evidence of primary malignancy or other sites of disease, and laboratory workup was otherwise normal. Computed tomography (CT)-guided core needle biopsy was performed but was non-diagnostic. Open biopsy was then pursued and demonstrated lymphocytes, plasma cells, and histiocytes with focal emperipolesis. Immunohistochemical stains for S100 were positive, consistent with primary RDD of the bone. After discussing options with a multidisciplinary tumor board, the lesions were treated with open curettage and bone grafting. The patient did well initially with complete resolution of pain, but unfortunately, his discomfort returned several months later. On follow-up MRI, residual disease was noted in two focal areas located at the periphery of the previously treated site. Due to the impossibility of visualizing this lesion with plain radiography, and thus a concern for a positive margin with further surgery, repeat excision was not advised. A tumor board was convened again, and the decision was made to pursue local radiation to the site. He underwent 36 Gray (Gy) radiation to the site (over 36 fractions) but unfortunately separated from the military service a few weeks later and was lost to follow-up.

Case 4

**Figure 4:**
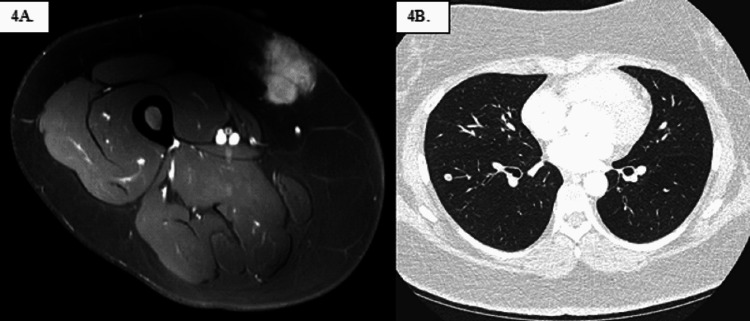
Lung involvement of Rosai-Dorfman disease of the right thigh Magnetic resonance imaging (MRI) with and without contrast (axial view) showed a superficial, solid, contrast-enhancing mass within the right medial thigh, with irregular borders (A). Computed tomography of the chest demonstrated a right lower lobe solitary, non-calcified 8 mm nodule concerning for metastasis of Rosai-Dorfman disease (B).

A 43-year-old female with a history of hypertension initially presented due to a progressive enlargement of a right upper medial thigh soft tissue mass over a one-year period. The mass was tender to palpation, and she was experiencing ipsilateral groin discomfort. The patient denied fevers, night sweats, weight loss, or a history of preceding trauma. Physical examination revealed a firm, mobile, subcutaneous mass on the superficial medial aspect of the right upper thigh with irregular borders. There were no overlying skin changes or palpable adenopathy. Soft tissue ultrasound of the right thigh mass showed an irregular infiltrative hypoechoic subcutaneous mass measuring 3.8 centimeters (cm) by 1.8 cm, which was confirmed on MRI imaging (Figure 4A). Computed tomography of the chest with contrast was performed due to the concern for a possible sarcoma, revealing a right lower lobe 8-millimeter (mm) nodule suspicious for metastasis (Figure 4B). A navigational bronchoscopy with tissue biopsy was negative for malignancy. Thereafter, an initial ultrasound-guided core needle biopsy of the right medial thigh mass demonstrated fibroadipose tissue with chronic inflammation and was also benign. After discussing the risks, benefits, and indications of surgical intervention, the patient opted to proceed with the excision of the right medial thigh mass due to her symptoms, and the excisional biopsy revealed pathology consistent with RDD and in the absence of other systemic findings, a diagnosis of cutaneous RDD was made. Serial radiographic observation postoperatively with MRI showed no evidence of recurrence or distant spread at her one-year postoperative appointment. The patient remains asymptomatic at this time and will continue clinical surveillance annually.

Case 5

**Figure 5:**
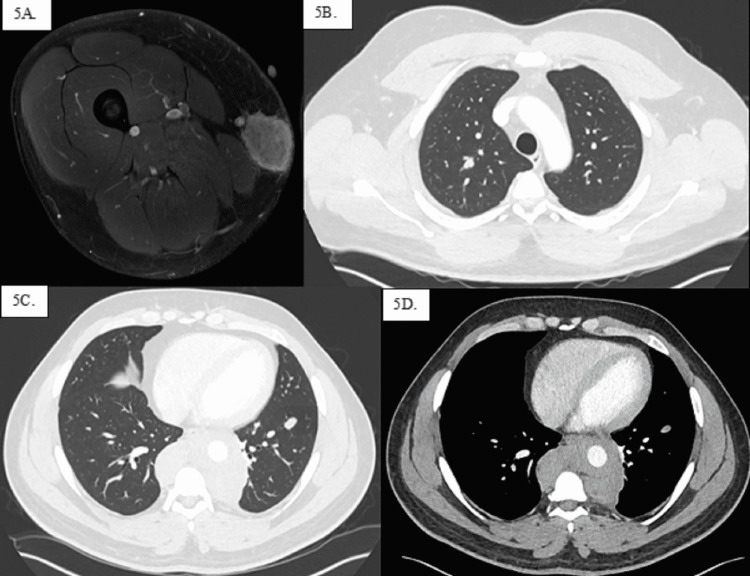
Case 5: Rosai-Dorfman disease systemic involvement A. Magnetic resonance imaging (MRI) with and without contrast showed a 5.1 cm homogenously contrast-enhancing mass within the right medial thigh (axial view). B. Computed tomography (CT) of the chest demonstrated a right upper lobe 1.2 cm non-calcified nodule. C. CT of the chest demonstrated a left lower lobe 1.3 cm solid, non-calcified nodule. D. CT of the chest showed a 10.6 x 9 cm homogenous, confluent paraspinal and posterior mediastinal soft tissue mass that surrounds the aorta without compression, which was concerning for metastatic disease.

A 30-year-old male on active duty in the Navy presented for a medial right thigh mass that appeared suddenly nine months prior. He denied any associated pain, inflammation, or skin changes, but did state he intermittently experienced regional numbness. The patient denied any constitutional symptoms. The mass had not changed in size over this time period. On physical exam, the patient had a 6 x 5 cm right medial thigh mass without associated skin changes, neurovascular deficits, or adenopathy. MRI of the right medial thigh demonstrated a 5.1 cm enhancing mass in subcutaneous tissue suggestive of sarcoma (Figure 5A). A CT of the chest for complete staging showed a 1.2 cm nodule in the right upper lobe, a 1.3 cm nodule in the left lower lobe, and a 10.6 x 9 cm posterior mediastinal mass encasing the thoracic aorta, concerning for metastatic disease (Figures 5B-5D). A core needle biopsy of the right medial thigh mass was non-diagnostic and thus an open biopsy was performed concurrently with an endoscopic ultrasound-guided biopsy of the paraaortic mass, both of which revealed histologic features most consistent with Rosai-Dorfman disease. Given the multi-focal nature and the lack of spontaneous resolution, systemic therapy was pursued with prednisone 60 mg daily for two months with a subsequent taper and surveillance imaging. Following corticosteroid therapy, the patient reported an interval decreased size of his right thigh lesion, however, this improvement plateaued and interval MRI imaging was stable. The chest lesions were unaltered. Tissue samples were sent for next-generation sequencing to assess for BRAF and MEK pathway mutations to guide treatment, and the patient was subsequently started on the MAPK1 inhibitor cobimetinib 60 milligrams daily for days 1 through 21 of a 28-day cycle [6]. The patient underwent surveillance imaging with a chest CT thereafter, which showed no significant change in the nodal mass about the lower middle and posterior mediastinum with persistent, small, nodular opacities within the right upper lobe and left lower lobe.

## Discussion

Rosai-Dorfman disease, also called sinus histiocytosis with massive lymphadenopathy, is a very rare disorder characterized by the abnormal proliferation and accumulation of histiocytes and was recognized as a distinct clinical entity in 1965 [5]. Most often, the disease occurs in younger individuals with a median age of diagnosis of approximately 20 years old and presents as bulky, painless cervical adenopathy with associated constitutional symptoms [5]. It has a slight predominance in males and those of African descent [5]. The literature suggests an incidence of only 100 cases per year in the United States [2]. Due to the clinical rarity, a specific inciting etiology has yet to be identified, and combined with the vast range of clinical manifestations, the early recognition, diagnosis, and initiation of effective therapy are especially challenging [2,7]. Of the reports documented, many cases of RDD have a self-limited course, particularly when the disease phenotype manifests as nodal or integument involvement [2]. However, approximately half of the patients present with extranodal disease, which is less likely to be self-limiting and may require intervention. The patients described in this case series illustrate the phenotypic heterogeneity of RDD as well as the unique treatment approaches based on the organ systems involved. To our knowledge, there is no existing report that describes such a high incidence at one institution of a cohort of patients with RDD, all within a period of 12 months.

Recognizing and diagnosing RDD is difficult in the context of its wide range of clinical manifestations and the ability to affect any organ group. The diagnosis of RDD hinges on biopsy and histopathologic evaluation, with several characteristic findings such as ample pale cytoplasm and enlarged histiocytes demonstrating emperipolesis [2,5]. Emperipolesis is the presence of intact leukocytes within the cytoplasm of another cell, which is suggestive albeit not pathognomonic for RDD. Extranodal variants are less likely to exhibit this pathologic feature [2,8], and it is seen in other histiocytic disorders. The enlarged histocytes are often surrounded by an inflammatory background of plasma cells and neutrophilic infiltrates [2]. Immunophenotyping can support the diagnosis and typically reveals S-100, fascin, and CD68 positivity in the affected histiocytes, but stains negatively for CD1a and CD207, unlike Langerhans cell histiocytosis [2,8-10]. Further complicating the recognition of this disease is the lack of understanding of the pathogenesis. It has been theorized that autoimmune disorders or viruses, such as human herpesvirus 6, Epstein-Barr virus infection, and human immunodeficiency virus (HIV), may be inciting etiologies; however, this has not been clearly demonstrated [2,11]. Interestingly, there have been more recent reports describing molecular pathway dysfunction involving the mitogen-activated protein kinase - extracellular signal-regulated kinase (MAPK-ERK) pathway [6,12]. This pathway includes the BRAF V600E and MAP2K1 mutations, which may be present in up to one-third of cases and confer candidacy for targeted therapy to MEK (MAPK) inhibition (currently being employed in Case 5) [6,12]. However, this has not been proven in all cases of RDD and further studies are required for elucidation but could represent an important implicated molecular pathway.

All of the cases described herein are unusual manifestations of an already rare disease. Three of the five cases included in this series describe isolated involvement of the bone and/or soft tissue, which occurs in less than 5-10% of RDD cases, as a nodal disease is also typically present in these cases [2,3] but was absent in ours. The incidence of primary osseous RDD is not known. Bone pain and swelling are frequent presenting complaints, and lesions are typically lytic, although mixed lytic and sclerotic lesions can be seen [2,3]. Purely sclerotic lesions are exceedingly rare [3]. Primary osseous RDD has been described as occurring in the metaphysis or diaphysis of long bones but is more frequently present in the cranium [2,3]. Optimal treatment has yet to be fully defined, however, surgical resection or curettage appears to be the mainstay of treatment for primary osseous involvement, and it may be theorized that nodal involvement may require a more systemic approach [3].

Moreover, the cases in this series occurred within a 12-month period in the same geographic area and at the same institution, provoking the question of whether a common environmental predisposing factor was present. This locoregional pattern without any prior relationship of any of the patients suggests a possible acquired somatic mutation from an environmental source, which thereby induced the development of atypical manifestations of RDD, although this is speculative. It is suggested in the literature that the pathogenesis is likely as heterogenetic as the disease itself, and there has not been a clearly established causation for the various phenotypic presentations of organ involvement. The sporadic high incidence in our region highlights the lack of concrete knowledge on the pathogenesis of this disease and which therapeutic modalities will decrease recurrence, improve survival, or reduce morbidity. Treatment for individuals with the extranodal disease at this time is largely individualized based on the site(s) of involvement. Corticosteroids, surgery, immunomodulating therapy, radiotherapy, chemotherapy agents, and now targeted molecular therapy have all been employed in cases where spontaneous resolution has not occurred. The ideal patient and exact niche of the available therapeutic modalities are still unclear, and the duration of therapy and surveillance is largely left to expert opinion and multidisciplinary discussion.

## Conclusions

Our series of contemporary cases describe a sizable cohort that highlights unique, atypical manifestations and treatment approaches to this exceptionally rare disease. Each of these cases illustrates unique obstacles to diagnosing and treating RDD, as each patient had a different treatment course and outcome. In doing so, the cases herein add valuable insight and knowledge to the scant published data of this clinical entity. A question of environmental predisposition is raised in our series, given the uncharacteristically high incidence of RDD in our population in such a short period of time. This may be indicative of a novel etiology of this disease, and further investigation is warranted. More data and molecular studies are needed to further elucidate possible etiologies and precipitating factors that contribute to the development of RDD, as well as follow up after initiation of therapies (i.e. targeted molecular treatments) to better assess the efficacy of the various treatments in current practice.
